# Association of sleep disturbances with sarcopenia and its defining components: the ELSA-Brasil study

**DOI:** 10.1590/1414-431X2021e11539

**Published:** 2021-12-03

**Authors:** C. Szlejf, C.K. Suemoto, L.F. Drager, R.H. Griep, M.J.M. Fonseca, M.F.H.S. Diniz, P.A. Lotufo, I.M. Benseãor

**Affiliations:** 1 Centro de Pesquisa Clínica e Epidemiológica, Hospital Universitário, Universidade de São Paulo, São Paulo, SP, Brasil; 2 Divisão de Geriatria, Faculdade de Medicina, Universidade de São Paulo, São Paulo, SP, Brasil; 3 Unidade de Hipertensão, Instituto do Coração, Hospital das Clínicas, Faculdade de Medicina, Universidade de São Paulo, São Paulo, SP, Brasil; 4 Laboratório de Educação em Ambiente e Saúde, Instituto Oswaldo Cruz, Fundação Oswaldo Cruz, Rio de Janeiro, RJ, Brasil; 5 Departamento de Epidemiologia e Métodos Quantitativos em Saúde, Fundação Oswaldo Cruz, Rio de Janeiro, RJ, Brasil; 6 Faculdade de Medicina, Universidade Federal de Minas Gerais, Belo Horizonte, MG, Brasil; 7 Departamento de Medicina Interna, Faculdade de Medicina, Universidade de São Paulo, São Paulo, SP, Brasil

**Keywords:** Body composition, Insomnia, Muscle strength, Obstructive sleep apnea, Sarcopenia

## Abstract

Sarcopenia and sleep problems share common physiopathology. We aimed to investigate the association of sleep disturbances with sarcopenia and its defining components in Brazilian middle-aged and older adults. In this cross-sectional analysis of the second wave of the ELSA-Brasil study, we included data from 7948 participants aged 50 years and older. Muscle mass was evaluated by bioelectrical impedance analysis and muscle strength by hand-grip strength. Sarcopenia was defined according to the Foundation for the National Institutes of Health criteria. Sleep duration and insomnia complaint were self-reported. Short sleep duration was considered as ≤6 h/night and long sleep duration as >8 h/night. High risk of obstructive sleep apnea (OSA) was assessed using the STOP-Bang questionnaire. Possible confounders included socio-demographic characteristics, lifestyle, clinical comorbidities, and use of sedatives and hypnotics. The frequencies of sarcopenia, low muscle mass, and low muscle strength were 1.6, 21.1, and 4.1%, respectively. After adjustment for possible confounders, high risk of OSA was associated with low muscle mass (OR=2.17, 95%CI: 1.92-2.45). Among obese participants, high risk of OSA was associated with low muscle strength (OR=1.68, 95%CI: 1.07-2.64). However, neither short nor long sleep duration or frequent insomnia complaint were associated with sarcopenia or its defining components. In conclusion, high risk of OSA was associated with low muscle mass in the whole sample and with low muscle strength among obese participants. Future studies are needed to clarify the temporal relationship between both conditions.

## Introduction

Sleep is essential for maintaining health and quality of life. Despite physiologic changes in sleep-awake patterns and sleep architecture during the aging process, older adults suffer with the consequences of a constellation of problems related to sleep duration, difficulty in initiating and maintaining sleep, and sleep-disordered breathing (1). The presence of sleep disturbances may trigger several pathways such as sympathetic activation, metabolic disturbances, and pro-inflammatory status (2), some of which are also implicated in the genesis of sarcopenia (3). Recently defined as a muscle failure, sarcopenia is characterized by the combination of low muscle strength with low muscle mass or altered muscle quality (4), increasing the risk of mortality, hospitalization, falls, fractures, and disability (5). As sarcopenia and sleep disturbances share common pathophysiology, investigating their association could improve the comprehension of involved mechanisms.

Previous works examined the relationship of sarcopenia components with sleep problems, finding associations of lower muscle mass with long sleep duration and poor sleep quality (6-9). Likewise, studies showed that lower grip strength was associated with longer sleep h (7), poorer sleep quality (10), and insomnia (11). However, the association of sleep problems with the current definition of sarcopenia, which combines low muscle strength and low muscle mass, was rarely studied. Hu et al. (12) found that among community-dwelling older adults, women with short and long sleep durations had a higher risk of sarcopenia defined according to the Asian Working Group for Sarcopenia. Moreover, few studies investigated the association of sarcopenia and obstructive sleep apnea (OSA), with conflicting findings (13-16). Therefore, we aimed to investigate the association of sarcopenia and its defining components with sleep duration, frequent insomnia complaint, and high risk of OSA in community-dwelling middle-aged and older adults participating in the ELSA-Brasil study.

## Material and Methods

### Study population and design

The present study is a cross-sectional analysis of the second wave of ELSA-Brasil, which was conducted after four years of baseline, between 2012 and 2014. The ELSA-Brasil is a cohort of active and retired employees from public institutions located in six Brazilian cities, aged between 35 and 74 years at baseline, with the aim to investigate cardiovascular diseases and diabetes in Brazilian adults. The study design and cohort profile have been published elsewhere (17). Of the 15,105 participants included at baseline, 14,104 were reassessed in the second data collection. Information was collected on socio-demographics, lifestyle factors, mental health, cognitive status, occupational exposure, anthropometric measurements, body composition, and laboratory tests. The study was conducted in accordance with the Declaration of Helsinki and was approved by the local institutional review boards. All participants signed the informed consent prior to enrollment. For this analysis, we excluded participants: 1) that were younger than 50 years at the time of the second data collection; 2) with self-reported weakness in both hands due to pain or any other condition; and 3) with incomplete data on exposure, outcomes, or covariates.

### Physical measurements and sarcopenia assessment

Anthropometric and body composition evaluations followed an overnight fast of 12 h. Height (in m) was measured with a fixed stadiometer with 0.1 cm accuracy (seca 216, seca, Brazil), weight (in kg) was measured with an electronic digital scale (Toledo Brasil Indústria de Balanças Ltda., Brazil), and body mass index (BMI) was calculated as weight divided by squared height (kg/m^2^). Obesity was defined as BMI ≥30 kg/m^2^. Body composition was assessed with a tetrapolar vertical bioelectrical impedance analyzer using 8-point tactile electrodes (Inbody230, InBody Co., Ltd., Korea). Bioelectrical impedance analysis is a simple, safe, accessible, and precise method to assess skeletal muscle mass in large studies, with accurate estimates compared to dual-energy X-ray absorptiometry (18). Muscle mass was assessed by appendicular lean mass standardized by BMI (ALM_BMI_), which is the sum of lean mass in arms and legs (in kg) divided by BMI. Muscle strength was examined with handgrip strength, using a Jamar^®^ hydraulic hand dynamometer (Sammons Preston, USA). Three measurements were taken from each hand, the highest of which was considered. We defined sarcopenia according to the Foundation for the National Institutes of Health (FNIH) Sarcopenia Project criteria, a data-driven operational definition validated to predict clinical outcomes. Sarcopenia was considered when the ALM_BMI_ was <0.789 for men and <0.512 for women, and handgrip strength was <26 kg for men and <16 kg for women (19).

### Sleep assessment

Sleep duration was assessed by the self-reported question “How many hours do you usually sleep at night?”. Participants were classified into short sleep duration (≤6 h), adequate sleep duration (>6 and ≤8 h), and long sleep duration (>8 h). Three questions examined subjective insomnia complaints: “In the past four weeks regarding your sleep at home at night, how often have you had difficulty falling asleep?”, “How often have you woken up and had difficulty falling asleep again?”, and “How often have you woken up before the desired time and not managed to fall asleep again?”. Participants answered using the following Likert-type scale: always, almost always, sometimes, rarely, and never. A frequent insomnia complaint was considered when participants answered “always” or “almost always” to at least one question.

OSA risk was assessed using the STOP-Bang questionnaire (20), a self-reported screening tool validated in Brazilian Portuguese (21), which includes the following items: snoring, tiredness, observed apnea, high blood pressure, BMI, age (older than 50 years), neck circumference, and gender. High risk of OSA was considered when the answer was yes to 5 or more items, or to at least 2 items among the STOP items, combined with one of the following: male, BMI >35 kg/m^2^, or neck circumference >43 cm for men and >41 cm for women (22). High blood pressure was defined as the use of anti-hypertensive drugs, systolic blood pressure ≥140 mmHg, or diastolic blood pressure ≥90 mmHg.

### Sociodemographic characteristics and clinical profile

Questionnaires addressed sociodemographic characteristics and lifestyle, such as age, gender, self-reported race (white, black, brown, other), education level (high school or lower *vs* higher education), current smoking status, and current alcohol consumption. Leisure-time physical activity was assessed by the International Physical Activity Questionnaire - long form (classifying participants as active, insufficiently active, and inactive) (23). Diabetes mellitus was considered based on self-reported information, use of oral hypoglycemic agents or insulin therapy, fasting plasma glucose ≥126 mg/dL, 2-h post-prandial 75 g glucose test ≥200 mg/dL, or glycated hemoglobin ≥6.5%. Depression was assessed by the Brazilian version of the Clinical Interview Scheduled Revised (24). The use of sedative and hypnotic drugs was recorded when participants reported current use of the following drug classes: benzodiazepines, anticonvulsants, neuroleptics, sedative antidepressants, antihistamines, opioids, and sleep inducers.

### Statistical analysis

Continuous variables with normal distribution are reported as means±SD, non-normally distributed continuous variables as median and interquartile range (IQR), and categorical variables as absolute and relative frequencies. Characteristics of participants according to sarcopenia were compared using the Student's *t*-test, Wilcoxon rank-sum test, chi-squared test, and Fisher's exact test for normally distributed continuous variables, continuous variables non-normally distributed, categorical variables, and categorical variables with small cell counts, respectively.

We used logistic regression models to investigate the association of short and long sleep duration, frequent complaint of insomnia, and high risk of OSA with sarcopenia, low muscle mass, and low muscle strength. Multiple models were initially adjusted for sociodemographic characteristics, such as age, gender, race, and education (model 1). Further model adjustment (model 2) differed based on the explanatory variable as follows: for sleep duration and insomnia complaint we included lifestyle factors (current smoking status, current alcohol use, and leisure-time physical activity), depression, and the use of sedatives and hypnotics; for risk of OSA we excluded sex and included lifestyle factors, diabetes mellitus, and the use of sedatives and hypnotics. Since short sleep duration and insomnia are risk factors for diabetes, we considered diabetes as a possible mediator (and not a confounder) on the association of sleep duration and insomnia complaint with sarcopenia; therefore, we did not adjust this analysis for diabetes. Using the same rationale, depression was not included in the adjustment of the models with OSA.

We also performed logistic regression using the STOP-Bang score as a continuous variable to investigate a dose-response association with sarcopenia and its defining components. Additionally, we investigated the independent association of each STOP-Bang item with sarcopenia and its defining components. Finally, because obesity is a condition closely related to sarcopenia and OSA (4,25), we tested its interaction with high risk of OSA for the associations with sarcopenia, low muscle mass, and low muscle strength. We stratified the analyses if an interaction term had a P-value <0.10. Data were analyzed using Stata 14.2 (StataCorp LP, USA). The alpha-level was set at 0.05.

## Results

**Figure 1:**
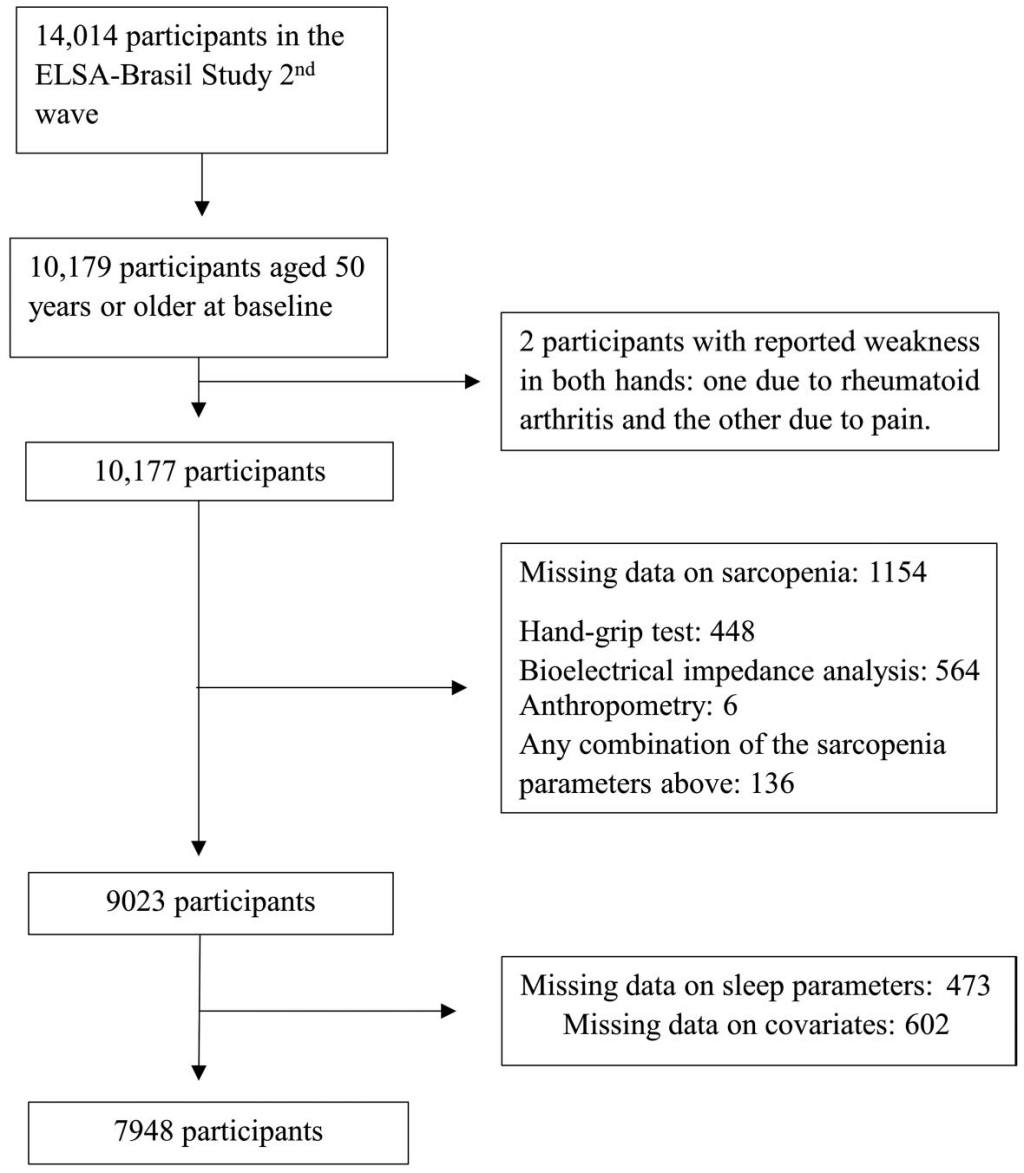
Study flowchart.

Among the 14,014 participants in the second wave of the ELSA-Brasil study, 7,948 fulfilled the criteria for the present study (mean age 59.5±7.0 years, 23.4% aged 65 years and older, 53.7% women) (Figure 1). Sarcopenia was present in 128 participants (1.6%). The frequencies of low muscle mass and low muscle strength were 21.1 and 4.1%, respectively. Additionally, frequencies of short sleep duration, long sleep duration, frequent insomnia complaint, and high risk of OSA were 49.2, 4.0, 21.3, and 25.2%, respectively. Participants' characteristics according to sarcopenia are shown in Table 1. Sarcopenic individuals were older, took more sedatives and hypnotics, had higher BMI, were more likely to have high risk of OSA, and were more likely to have depression, diabetes mellitus, and insomnia complaint. They also had lower education level and lower alcohol consumption.

**Table 1:** Characteristics of study participants according to sarcopenia (n=7948).

	Without sarcopenia (n=7820)	With sarcopenia (n=128)	P
Sleep duration, n (%)			0.261^†^
Short (≤6 h)	3836 (49.1)	72 (56.3)	
Long (>8 h)	315 (4.0)	5 (3.9)	
Frequent insomnia complaint, n (%)	1928 (24.7)	45 (35.2)	0.006^‡^
High risk of obstructive sleep apnea (STOP-Bang)	1968 (25.2)	43 (33.6)	0.030^‡^
Age (years), mean (SD)	59.4 (6.9)	65.7 (8.0)	<0.001^§^
Female, n (%)	4190 (53.6)	79 (61.7)	0.067^‡^
Race, n (%)			0.346^†^
Black	1169 (15.0)	13 (10.1)	
Brown	2019 (25.8)	37 (28.9)	
White	4316 (55.2)	71 (55.5)	
Other	316 (4.0)	7 (5.5)	
Higher education, n (%)	4329 (55.4)	55 (43.0)	0.005^‡^
Body mass index, mean (SD)	27.7 (4.8)	29.5 (5.1)	<0.001^§^
Obesity, n (%)	53 (41.4)	2104 (26.9)	<0.001^‡^
Current smoker, n (%)	2796 (35.8)	44 (34.4)	0.747^‡^
Current alcohol consumption, n (%)	5100 (65.2)	63 (49.2)	<0.001^‡^
Leisure-time physical activity, n (%)			0.007^†^
Inactive	4603 (58.9)	91 (71.1)	
Insufficiently active	1110 (14.2)	17 (13.3)	
Active	2107 (26.9)	20 (15.6)	
Depression, n (%)	347 (4.4)	12 (9.4)	0.015^†^
Use of sedatives or hypnotics, n (%)	1373 (17.6)	32 (25.0)	0.029^‡^
Diabetes mellitus, n (%)	1953 (25.0)	57 (44.5)	<0.001^‡^

SD: standard deviation; ^†^Fisher's exact test; ^‡^chi-squared test; ^§^two sample *t*-test.

Table 2 shows the association of sleep duration with sarcopenia and its defining components. Neither short nor long sleep duration was associated with sarcopenia, low muscle mass, and low muscle strength, either in a crude analysis or after adjustment for sociodemographic characteristics, lifestyle factors, depression, and the use of sedatives and hypnotics. Frequent insomnia complaint was associated with sarcopenia and low muscle strength in crude analysis, although the association was no longer significant in the fully adjusted model (see Table 3).

**Table 2:** Association of sleep duration with sarcopenia and its defining components (n=7948).

	Sarcopenia		Low muscle mass		Low muscle strength	
	OR (95%CI)	P	OR (95%CI)	P	OR (95%CI)	P
Short sleep duration (≤6 h)						
Crude	1.35 (0.94-1.94)	0.104	1.17 (1.05-1.31)	0.006	1.07 (0.85-1.34)	0.586
Model 1^†^	1.41 (0.97-2.04)	0.068	1.13 (1.01-1.27)	0.033	1.15 (0.91-1.45)	0.244
Model 2^‡^	1.32 (0.91-1.92)	0.150	1.12 (1.00-1.26)	0.054	1.10 (0.87-1.39)	0.437
Long sleep duration (>8 h)						
Crude	1.14 (0.45-2.88)	0.779	1.36 (1.05-1.78)	0.022	1.29 (0.76-2.19)	0.348
Model 1^†^	0.96 (0.38-2.44)	0.929	1.19 (0.90-1.56)	0.224	1.17 (0.68-2.01)	0.563
Model 2^‡^	0.90 (0.35-2.31)	0.828	1.16 (0.88-1.52)	0.307	1.11 (0.65-1.91)	0.705

^†^Model 1: logistic regression adjusted for age, gender, race, and education. ^‡^Model 2: logistic regression adjusted for age, sex, race, education, leisure-time physical activity, current smoker, current alcohol intake, depression, and current use of sedatives or hypnotics.

**Table 3:** Association of frequent insomnia complaint with sarcopenia and its defining components (n=7948).

Frequent complaint of insomnia	Sarcopenia		Low muscle mass		Low muscle strength	
	OR (95%CI)	P	OR (95%CI)	P	OR (95%CI)	P
Crude	1.66 (1.15-2.39)	0.007	1.10 (0.97-1.24)	0.128	1.39 (1.09-1.76)	0.008
Model 1^†^	1.47 (1.01-2.14)	0.043	1.04 (0.92-1.19)	0.514	1.28 (1.00-1.64)	0.048
Model 2^‡^	1.28 (0.87-1.88)	0.218	1.03 (0.90-1.17)	0.693	1.14 (0.89-1.48)	0.306

^†^Model 1: logistic regression adjusted for age, gender, race, and education. ^‡^Model 2: logistic regression adjusted for age, sex, race, education, leisure-time physical activity, current smoker, current alcohol intake, depression, and current use of sedatives or hypnotics.

The association of high risk of OSA with sarcopenia and its defining components is shown in Table 4. Participants with high risk of OSA had increased odds of low muscle mass after adjustment for possible confounders, although high risk of OSA was not associated with sarcopenia or low muscle strength. Additionally, higher STOP-Bang scores were associated with higher odds of low muscle mass, but not with sarcopenia or low muscle strength (Supplementary Table S1). When we tested interactions between high risk of OSA and obesity, we found that it was significant for the association with low muscle strength (interaction term P-value=0.018), while it was not significant for the associations with sarcopenia (interaction term P-value=0.315) and low muscle mass (interaction term P-value=0.913). Accordingly, when we stratified the analysis based on obesity, high risk of OSA was associated with low muscle strength only among obese participants and the measures of association of high risk of OSA with low muscle mass were similar between obese and non-obese participants (Table 5).

**Table 4:** Association of high risk of obstructive sleep apnea with sarcopenia and its defining components (n=7948).

	Sarcopenia		Low muscle mass		Low muscle strength	
	OR (95%CI)	P	OR (95%CI)	P	OR (95%CI)	P
Crude	1.51 (1.04-2.19)	0.029	2.43 (2.17-2.72)	<0.001	1.07 (0.83-1.38)	0.597
Model 1^†^	1.37 (0.94-2.00)	0.097	2.33 (2.07-2.63)	<0.001	1.00 (0.78-1.30)	0.970
Model 2^‡^	1.29 (0.88-1.90)	0.195	2.17 (1.92-2.45)	<0.001	1.01 (0.78-1.32)	0.918

Risk of obstructive sleep apnea was assessed by the STOP-Bang questionnaire. ^†^Model 1: logistic regression adjusted for age, race, and education. ^‡^Model 2: logistic regression adjusted for age, race, education, leisure-time physical activity, current smoker, current alcohol intake, current use of sedatives or hypnotics, and diabetes mellitus.

**Table 5:** Association of high risk of obstructive sleep apnea with sarcopenia and its defining components according to obesity status.

	Sarcopenia		Low muscle mass		Low muscle strength	
	OR (95%CI)	P	OR (95%CI)	P	OR (95%CI)	P
Obese (BMI ≥30 kg/m^2^), n=2157						
Crude	1.35 (0.79-2.34)	0.275	1.63 (1.37-1.94)	<0.001	1.49 (0.96-2.30)	0.074
Model 1^†^	1.45 (0.83-2.51)	0.189	1.69 (1.42-2.02)	<0.001	1.56 (1.00-2.42)	0.049
Model 2^‡^	1.56 (0.89-2.74)	0.125	1.68 (1.40-2.02)	<0.001	1.68 (1.07-2.64)	0.024
Non-obese (BMI <30 kg/m^2^), n=5791						
Crude	1.25 (0.72-2.15)	0.425	1.99 (1.68-2.35)	<0.001	0.92 (0.66-1.29)	0.622
Model 1^†^	0.96 (0.55-1.66)	0.874	1.72 (1.44-2.06)	<0.001	0.78 (0.56-1.10)	0.161
Model 2^‡^	0.87 (0.49-1.54)	0.632	1.64 (1.37-1.97)	<0.001	0.79 (0.55-1.11)	0.175

Risk of obstructive sleep apnea was assessed by the STOP-Bang questionnaire. ^†^Model 1: logistic regression adjusted for age, race, and education. ^‡^Model 2: logistic regression adjusted for age, race, education, leisure-time physical activity, current smoker, current alcohol intake, current use of sedatives or hypnotics, and diabetes mellitus.

Considering the independent associations of each STOP-Bang item, we found that tiredness and BMI ≥35 kg/m^2^ were associated with higher odds of sarcopenia, while neck circumference was associated with lower odds of sarcopenia; high blood pressure, BMI ≥35 kg/m^2^, and male gender were associated with higher odds of low muscle mass; tiredness was associated with higher odds of low muscle strength, while male gender was associated with lower odds of low muscle strength (Supplementary Table S2).

## Discussion

The present study is a novelty as it investigated the association between multiple sleep disturbances and sarcopenia defined according to recent consensus criteria in a large sample of middle-aged and older adults. We found that high risk of OSA and higher STOP-Bang scores were associated with low muscle mass, but not with sarcopenia and low muscle strength in the whole sample. We also found that among obese participants, high risk of OSA was associated with low muscle strength. In contrast, our study did not find an association between sleep duration or frequent complaint of insomnia and sarcopenia and its defining components.

Few studies investigated the relationship between sarcopenia or its components with OSA. Our findings were similar to those of Fernandes et al. (13) that showed that sarcopenia was not associated with OSA assessed by a wrist-worn portable device among non-dialyzed chronic kidney disease patients. In a recent Brazilian study that included middle-aged and older adults, OSA objectively measured by laboratory-based polysomnography was not associated with sarcopenia defined as low muscle mass according to the FNIH criteria, although it was associated with sarcopenic obesity, defined as low muscle mass combined with high total body fat (16). In contrast, community-dwelling older adults with sleep-disordered breathing assessed by portable polysomnography had higher risk of low grip strength and slow walking speed in a cross-sectional analysis of the Cardiovascular Health Study (14). In a cross-sectional study including adults submitted to a comprehensive health examination, participants with reported daytime sleepiness combined with snoring and apnea had lower handgrip strength (26). In our study, the association of high risk of OSA with low muscle strength was only present in obese participants, possibly reflecting the negative effect of obesity on muscle function (27).

Our findings of sleep duration are in contrast with many studies, which demonstrated that individuals who sleep longer have lower muscle mass (6-9) and that long sleep duration predicts low muscle strength (7). Differences in the measurement of muscle mass and strength and in adopted cutoffs may partially explain the discrepancy. Also contrary to our study, Auyeung et al. (11) demonstrated that subjective insomnia complaint was associated with weaker grip strength in community-dwelling older men. On the other hand, similar to our findings, a cross-sectional study did not find an association of sleep latency, a surrogate measure for initial insomnia, with sarcopenia defined according to the FNIH criteria (28).

Our study did not assess potential mechanisms, but several pathways could explain the association of high risk of OSA with low muscle mass. Both conditions are associated with a decrease in the signaling of the growth hormone/insulin-like growth factor axis, insulin resistance, diabetes, and metabolic syndrome (29). Evidence indicates that OSA treatment with continuous positive airway pressure significantly increases growth hormone and insulin-like growth factor secretion (30), as well as increasing lean mass in men younger than 60 years (31). Although it is not yet clear which OSA alterations might lead to low muscle mass, we hypothesized that chronic hypoxia and fragmented sleep harmfully influence the major neuroendocrine stress systems: the autonomic sympatho-adrenal system, and the hypothalamic-pituitary-adrenal axis (29,32). Physical inactivity, which is highly associated with OSA, could also explain an increased odds for low muscle mass. Above all, we must consider the probable bidirectional association, with individuals with low muscle mass having a higher risk of OSA due to changes in muscle quality, physical inactivity, concomitant obesity, and comorbidities.

Different aspects could explain the low prevalence of sarcopenia in our sample. The ELSA-Brasil study is a cohort of highly educated active and retired civil servants, whose life trajectories could have positively influenced muscle mass and function. Another relevant point is the high proportion of middle-aged adults in the sample, around 75%. The mean age of sarcopenic participants was around 6 years higher than that of the non-sarcopenic participants. Additionally, the definition of sarcopenia was based on the FNIH criteria, which adopt strict cutoff points and require the combination of low muscle mass and low muscle strength (19). The prevalence of sarcopenia did not differ according to sex and race in our sample. A recent meta-analysis of 41 studies with older adults from different regions of the world that assessed sarcopenia according to different consensus definitions also found similar prevalence among community-dwelling men and women (33). However, in a study including a large representative sample of older American adults, the prevalence of sarcopenia defined according to FNIH criteria for low muscle mass varied by gender and race (34).

Our study had several strengths. We defined sarcopenia according to recent consensus definitions that propose a combination of low muscle mass with low muscle strength (4,19). The FNIH criteria were based on a data-driven approach that selected muscle parameters associated with incident mobility impairment, a prognostic marker in older adults (35), based on diverse populations from multiple cohort studies, although recent work from the same consortium has raised uncertainties about the role of low muscle mass in defining sarcopenia (36). We also included a large range of sleep problems, and we adjusted the analysis for relevant confounders, such as sociodemographic characteristics, cardiovascular risk factors, lifestyle factors, depression, and the use of sedatives and hypnotics. Nevertheless, our study also presented several limitations. The cross-sectional design prevented establishing a temporal association between high risk of OSA and sarcopenia. Although the present analysis used information from the second data examination of the ELSA-Brasil study, we could not estimate the incidence of sarcopenia because sarcopenia parameters were not measured at baseline. Future analyses of upcoming examinations of the cohort may assess the evolution of sarcopenia in participants with high risk of OSA. Additionally, future studies replicating these findings in different populations are also needed, as our results rely on a large sample size and on multiple comparisons without correction. Another limitation was the use of subjective questionnaires to assess sleep disturbances. Self-reported sleep has moderate correlation with actigraphy measurements of sleep duration, and studies have shown that individuals usually report longer periods of sleep than the objectively measured time (37,38). This fact might explain the lack of association between sleep duration and sarcopenia. Although the STOP-Bang questionnaire is a valid and acceptable tool to screen individuals for OSA risk with reasonable sensitivity and specificity (20), we could not assess OSA and its severity objectively. Notwithstanding, sleep duration and OSA are being evaluated by actigraphy and a portable sleep monitor, respectively, in a subsample of the cohort, and future studies with more precise data on sleep parameters will be conducted. Additionally, we used bioelectrical impedance analysis to measure appendicular lean mass instead of dual energy X-ray absorptiometry, the technique to measure muscle mass proposed by the FNIH criteria (19). Also, we did not exclude participants with clinical conditions that could have influenced muscle parameters, such as neurological and pulmonary conditions. Finally, we adjusted the analysis for relevant confounders on the association between sleep problems and sarcopenia, but residual confounding cannot be ruled out.

In conclusion, high risk of OSA was associated with low muscle mass in Brazilian middle-aged and older adults, and it was associated with low muscle strength among obese participants. This finding could encourage future research to clarify the temporal relationship between these conditions, ultimately opening the possibility to treat OSA to improve sarcopenia parameters or vice-versa.

## Supplementary Material

Click to view [pdf].
